# LGR4 and LGR5 form distinct homodimers that only LGR4 complexes with RNF43/ZNRF3 to provide high affinity binding of R-spondin ligands

**DOI:** 10.1038/s41598-023-37856-w

**Published:** 2023-07-04

**Authors:** Yukimatsu Toh, Ling Wu, Soohyun Park, Allison Wang, Jianghua Tu, Wangsheng Yu, Mingxin Zuo, Kendra S. Carmon, Qingyun J. Liu

**Affiliations:** Center for Translational Cancer Research, The Brown Foundation Institute of Molecular Medicine, University of Texas Health Science Center at Houston, 1825 Pressler St., Suite 330E, Houston, TX 77030 USA

**Keywords:** Biochemistry, Stem cells

## Abstract

LGR4 and LGR5 are two homologous receptors that potentiate Wnt/β-catenin signaling in response to R-spondin (RSPO) ligands. The RSPO and LGR4 complex binds to and inhibits activities of two related E3 ubiquitin ligases, RNF43 and ZNRF3, and thus protects Wnt receptors from the E3 ligase-mediated degradation. The RSPO and LGR5 complex, however, does not interact with the E3 ligases, and the structural basis of this difference remained unknown. Here we examined the affinities of monovalent and bivalent RSPO ligands in binding to LGR4, RNF43/ZNRF3, and LGR5 in whole cells and found unique features among the receptors and E3 ligases. Monovalent RSPO2 furin domain had much lower affinity in binding to LGR4 or RNF43/ZNRF3 than the bivalent form. In contrast, monovalent and bivalent forms had nearly identical affinity in binding to LGR5. Co-expression of ZNRF3 with LGR4 led to much higher binding affinity of the monovalent form whereas co-expression of ZNRF3 with LGR5 had no effect on the affinity. These results suggest that LGR4 and RNF43/ZNRF3 form a 2:2 dimer that accommodates bivalent binding of RSPO whereas LGR5 forms a homodimer that does not. Structural models are proposed to illustrate how RSPOs bind to LGR4, RNF43/ZNRF3, and LGR5 in whole cells.

## Introduction

LGR4, LGR5, and LGR6 (Leucine-rich repeat-containing G-protein- coupled receptor 4, 5, and 6) are three related membrane receptors with pleotropic functions in organ development and tumorigenesis^1–5^. Loss-of-function mutations in *LGR4* in humans is associated with low bone density, abnormal female reproductive systems and other traits while knockout of *Lgr4* in the mouse led to developmental defects with phenotypes similar to those of human mutation^6–8^. Knockout of *Lgr5* in the mouse led to neonatal lethality due to tongue defects^9^. In adult tissues, *LGR4* is widely expressed in epithelial tissues, particularly in proliferating cells ^4,10,11^, whereas *LGR5* is expressed mostly in stem cells in the gastrointestinal tract and the skin^4,12^. Both *Lgr4* and *Lgr5* are expressed in mouse intestinal stem cells, yet only *Lgr4* is essential for the survival and self-renewal of the stem cells and the integrity of the intestine^13–15^. In some tissues, deletion of *Lgr5* alone had no effect but enhanced the effect of *Lgr4* knockout^15^, suggesting that *Lgr4* and *Lgr5* plays similar roles in organ development and tissue maintenance with *Lgr4* having a dominating role.

LGR4, LGR5 and LGR6 all bind R-spondins, a group of four secreted proteins (RSPO1-4) and potentiate Wnt/β-catenin signaling^13,16,17^. Mechanistically, RSPO and LGR4 has been shown to increase Wnt receptor levels and enhance signaling by binding to and inhibiting two E3 ubiquitin ligases RNF43/ZNRF3 that otherwise ubiquitinate Wnt receptors for degradation^18–20^. For LGR5, we showed that the RSPO and LGR5 complex interacts with the Wnt signalosome to enhance Wnt/β-catenin signaling without interacting with the E3 ligases^20^. The N-terminal furin domain of RSPOs contain two sub-domains, furin-1 (Fu1) and furin-2 (Fu2), and X-ray co-crystal structures revealed that Fu1 binds to the extracellular domain (ECD) of RNF43/ZNRF3 while Fu2 binds to the ECDs of LGR4 and LGR5^21–25^. Importantly, when crystalized in the absence of RNF43- or ZNRF3-ECD, RSPO furin domain and LGR5ECD formed structures that were not compatible with further binding to RNF43/ZNRF3-ECD whereas RSPO furin-LGR5ECD-RNF43ECD would form a ternary complex when crystalized together^24–26^. Here we examined the binding profiles and function of LGR4, LGR5, RNF43, and ZNRF3 in whole cells in parallel using monovalent and bivalent RSPO2 furin domain ligands. The findings have substantiated that LGR4 and LGR5 bind RSPOs in distinct patterns and that only LGR4 interacts with the E3 ligases.

## Results

### RSPO2 furin domain alone had low affinity for LGR4

**Figure 1:**
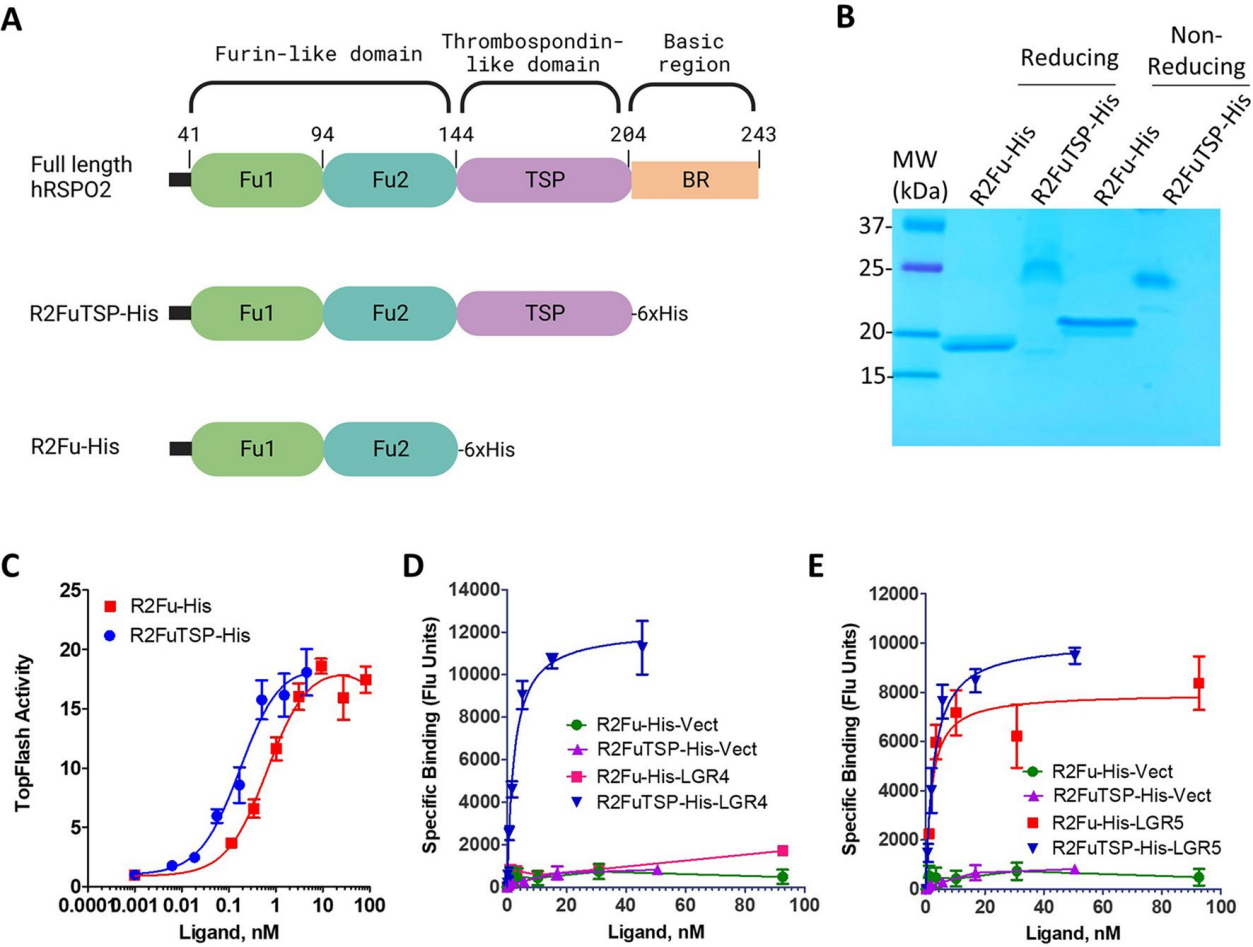
RSPO2 furin domain had low affinity for LGR4 but high affinity for LGR5. (**A**), Schematic diagram of RSPO2 domain structure and the two truncated forms analyzed here. (**B**), Coomassie blue staining of purified R2Fu-His and R2FuTSP-His under reducing and non-reducing conditions. (**C**), Dose-dependent increase of TOPFlash activity by R2Fu-His and R2FuTSP-His in STF cells. Assays were performed with a fixed concentration of Wnt3a and increasing concentrations of the indicated ligands. The activity was normalized to baseline (Wnt3a only). (**D**), Saturation binding of R2Fu-His and R2FuTSP-His to HEK293T cells over-expressing LGR4 or vector control. Specific binding was calculated by subtracting background fluorescence (no ligand present, only fluorescence-labeled antibody) from total fluorescence signal. (**E**), Binding of R2Fu-His and R2FuTSP-His to HEK293T cells expressing vector or LGR5ΔCT. Specific binding was as defined in D. Each graph is one representative of repeat experiments with similar conclusions. Each data point represents the mean of two or three replicates. Error bars are S.E.M.

Full-length RSPO2 contain three domains, a furin-like domain for RNF43 and LGR binding, and thrombospondin-like (TSP) domain and basic region for heparan sulfate proteoglycans (HSPGs) binding (Fig. 1A)^21,25,27^. To compare the function of RSPOs with and without the C-terminal thrombospondin (TSP) domain, we expressed and purified RSPO2 furin domain (R2Fu) with a C-terminal His tag (R2Fu-His) and verified its purity and monovalent nature (Fig. 1B). R2Fu-His was then compared with R2FuTSP-His (R2Fu with TSP domain) using Wnt/β-catenin signaling luciferase reporter SuperTopFlash (STF) cells^28^. STF cells endogenously express LGR4, RNF43, and ZNRF3 but no LGR5 and LGR6 at a functional level^16,18,29^. R2Fu-His was fully functional with a ~ 4 × lower in potency when compared with R2FuTSP-His (EC50 of 0.79 ± 0.08 nM vs. 0.18 ± 0.07 nM, Fig. 1C), consistent with the previous report of the importance of TSP domain in potency^27^. We then measured and compared the binding affinity of R2Fu-His and R2FuTSP-His to LGR4 and LGR5ΔCT using HEK293 cells over-expressing the receptors with fluorescence-labeled anti-His antibody. LGR5ΔCT, human LGR5 without the cytoplasmic C-terminal tail, is fully functional in Wnt/β-catenin signaling and was used here due to its reduced constitutive endocytosis and increased cell surface localization^30,31^. Receptor level of LGR5 wild type (LGR5WT) expressed on HEK293 cells is too low for binding affinity analysis using the fluorescence-based whole cell-binding assay^20,31^. For LGR4 cells, only low level binding could be detected with R2Fu-His while robust, high-affinity binding was observed with R2FuTSP-His (Kd = 2.2 ± 0.3 nM, Fig. 1D and Supplementary Fig. S2A). In contrast, similar high affinity binding of both R2Fu-His and R2FuTSP-His were observed on LGR5ΔCT cells (Kd = 1.9 ± 0.8 nM and 2.6 ± 0.5 nM, respectively, Fig. 1E and Supplementary Fig. S2B). Only negligible binding was detected on vector control cells (Fig. 1D,E), indicating that the binding was mediated by LGR4 or LGR5. As the TSP domain binds heparin sulfate proteoglycans (HSPG)^27^, these results suggest that RSPO2 furin domain alone has relatively low binding affinity to full-length LGR4 and that LGR4 interacts with HSPG, resulting in strong binding of R2FuTSP-His to LGR4-HSPG complexes. Alternatively, R2Fu-His binds to LGR4 in such a configuration that the His-tag was not detectable since R2Fu-His was fully active in the Wnt/β-catenin signaling assay (Fig. 1C) and we previously showed that bivalent IgG1-Fc-tagged RSPO furin domain binding to LGR4 was readily detected with high affinity and specificity^32^. In addition, the data also implies that HSPG has no effect on RSPO2 furin domain and LGR5 interaction since R2Fu-His and R2FuTSP-His has nearly the same affinity (1.9 ± 0.8 nM and 2.6 ± 0.5 nM, Fig. 1E).

### Bivalent and monovalent forms of RSPO2 furin domain show distinct binding and functional profiles on LGR4 but not on LGR5 cells

**Figure 2:**
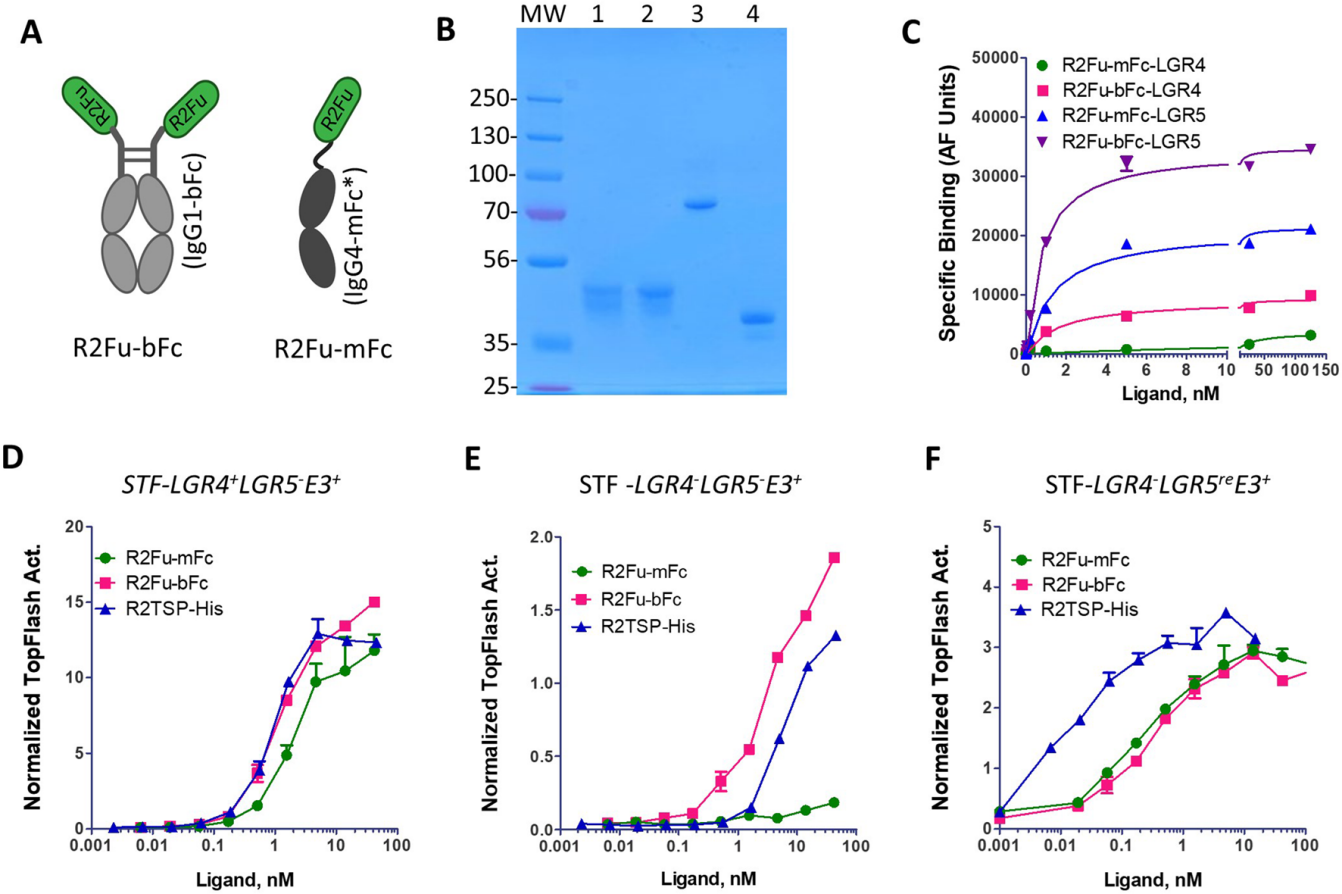
Monovalent and bivalent RSPO2 furin domains had distinct binding affinity on LGR4 but not on LGR5. (**A**), Schematic diagram of bivalent and monovalent R2Fu-Fc. R2Fu is colored green while the IgG-Fc is grey. (**B**), SDS-PAGE image of R2Fu-bFc and R2Fu-mFc. Approximately 2 ug of each protein was loaded with (reducing) or without (non-reducing) beta-mercaptoethanol treatment. Lanes 1 and 3, R2Fu-bFc; Lanes 2 and 4, R2Fu-mFc. Lanes 1–2, reducing; Lanes 3–4, non-reducing. MW = molecular weights markers in Kd. (**C**), Saturation binding of R2Fu-mFc and R2Fu-bFc to HEK293T cells expressing LGR4 or LGR5ΔCT. (**D**), TOPFlash activity of the indicated ligands in parental STF cells after normalization by cell number. (**E**), TOPflash activity of the indicated ligands in STF cells with KO of LGR4 after normalization by cell number. (**F**), TOPFlash activity of the indicated ligands in STF-LGR4KO cells with recombinant expression of LGR5WT after normalization by cell number. Each graph is one representative of repeat experiments with similar conclusions, and each data point is the mean of two or three replicates. Error bars are S.E.M.

Since bivalent R2Fu-Fc binding to LGR4 was readily detectable with high affinity^32^, we asked if this was due to bivalent nature of IgG1-Fc fusion proteins (Fig. 2A, left panel). To test this, we fused R2Fu to the N-terminus of a monovalent form of human IgG4-Fc (R2Fu-mFc) that contains dimerization-abolishing mutations and exists in monovalent form (Fig. 2A, right panel)^33^. In comparison, R2Fu fused to wild-type IgG1-Fc domain always exists as a dimer and thus bivalent form (R2Fu-bFc). Gel analysis confirmed that under non-reducing conditions, R2Fu-bFc and R2Fu-mFc behaved as dimers and monomers, respectively, whereas both were monomers under reducing conditions (Fig. 2B). The two ligands were then tested for binding to HEK293 cells expressing LGR4 or LGR5ΔCT side-by-side using the same fluorescence-labeled anti-Fc antibody. For LGR4, R2Fu-bFc showed a Kd of 1.6 ± 0.3 nM whereas R2Fu-mFc showed much lower binding with Kd > 50 nM (Fig. 2C and Supplementary Fig. S3). For LGR5, both R2Fu-mFc and R2Fu-bFc showed similar binding affinity (1.4 ± 0.3 vs. 0.8 ± 0.1 nM). These results indicated that bivalent R2Fu-bFc had much higher binding affinity to LGR4 but similar affinity to LGR5 when compared to the monovalent form, suggesting LGR4 and LGR5 had distinct configurations on the cell surface.

Next, we compared the activity of R2Fu-bFc and R2Fu-mFc in potentiating Wnt/β-catenin signaling. In parental STF cells, R2Fu-mFc was fully active with only a 2 × folder lower potency when compared R2Fu-bFc and R2FuTSP-His (Fig. 2D), similar to the profile of R2Fu-His. In STF cells with knockout (KO) of LGR4, monovalent R2Fu-mFc showed very low activity when compared to the bivalent form or the monovalent R2FuTSP-His (Fig. 2E). As LGR4 and the RNF43/ZNRF3 E3 ligases form a complex even without RSPO^20^, these results are consistent with a model that monovalent R2Fu-His and R2Fu-mFc can bind to the LGR4 and E3 ligase complex with an affinity that is ~ 2 × lower than the bivalent R2Fu form. The potent activity of R2Fu-bFc on STF-LGR4KO cells further suggests that the E3 ligases also exist as dimers on the cell surface that enables high affinity binding of bivalent R2Fu, consistent with the X-ray co-crystal structures of R2Fu and RNF43/ZNRF3-ECD^25^. In STF-LGR4KO cells with recombinant expression of LGR5WT^20^, R2Fu-mFc and -bFc had nearly identical potency, but much lower than that of R2FuTSP (Fig. 2F), suggesting that R2Fu binding to LGR5 is not increased by pre-dimerization of the ligand and TSP domain can further enhance the activity of R2Fu in Wnt/β-catenin signaling without affecting binding to LGR5.

### Bivalent and monovalent RSPO2 furin domain also showed distinct binding affinities to endogenously expressed LGR4 and LGR5

**Figure 3:**
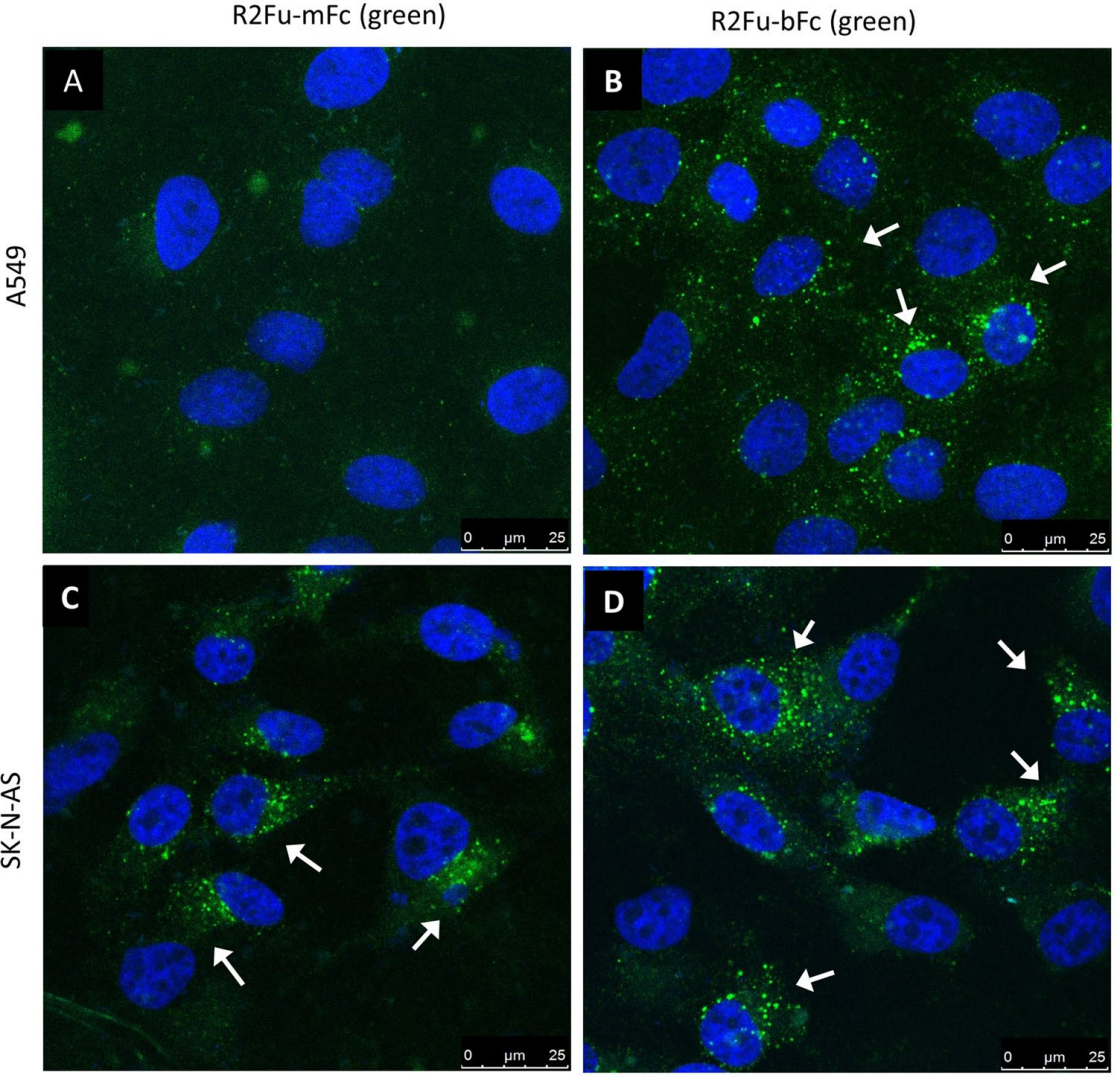
R2Fu-mFc and R2Fu-bFc had distinct binding profile in cells with endogenous expression of LGR4 but not in cells with endogenous expression of LGR5. (**A**-**B**), Confocal microscopy images of A549 cells incubated with R2Fu-mFc (**A**) or R2Fu-bFc (**B**). (**C**-**D**), Confocal microscopy images of SK-N-AS cells incubated with R2Fu-mFc (**C**) or R2Fu-bFc (**D**). In all images, live cells were incubated with the indicated ligands (125 nM) at 37 °C for 1 h, washed, fixed, permeabilized, and stained with Alexa488-labeled anti-human IgG. White arrows point to green punctates that represent endocytic vesicles containing RSPO ligands. Nuclei (blue) were stained with TO-PRO-3.

To confirm the binding profile difference of monovalent and bivalent RSPO2 furin domain forms, we examined the binding of the two forms on cancer cell lines that express either LGR4 or LGR5 endogenously. Previously, we showed that LGR4 was highly expressed in the lung cancer cell line A549 and RNA-seq expression data showed no expression of LGR5, LGR6, RNF43, and ZNRF3 in A549 cells^34^. R2Fu-mFc and -bFc were incubated with live A549 cells, followed by staining with fluorescence-labeled anti-human Fc. Confocal microscopy analysis revealed that R2Fu-mFc had no detectable binding while R2Fu-bFc showed strong, vesicle-enriched binding due to endocytosis (Fig. 3A,B). The neuroblastoma cell line SK-NA-S only expresses LGR5 as shown by us and others, and RNA-seq data showed no or little expression of LGR4, LGR6, RNF43, or ZNRF3^20,35^. Monovalent and bivalent R2Fu were incubated with live SK-N-AS cells and confocal analysis showed a similar level and pattern of binding for both forms (Fig. 3C,D). No endocytic fluorescence signal was observed in either cell line without added ligands. These results indicated that endogenously expressed LGR4 alone could only bind bivalent R2Fu with high affinity whereas LGR5 could bind either monovalent and bivalent form with high affinity, consistent with the observations made with recombinant, over-expressed receptors on HEK293T cells.

### RNF43 and ZNRF3 also exist as dimers on the cell surface

**Figure 4:**
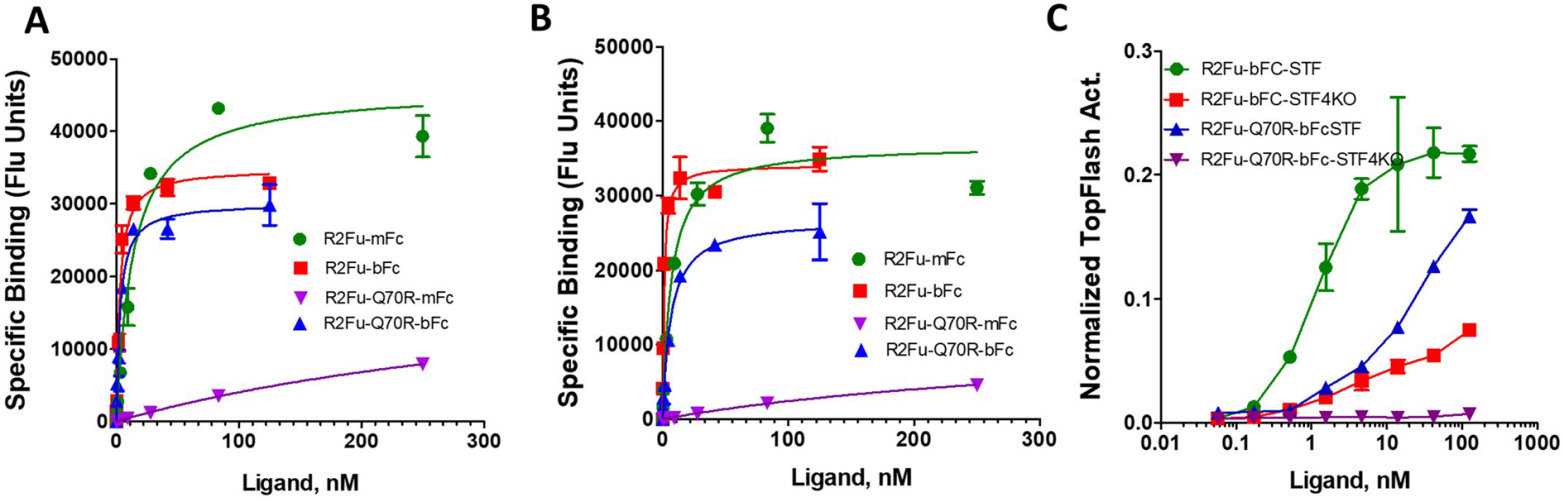
RNF43 and ZNRF3 also existed as dimers on the cell surface. (**A**), Saturation binding of the indicated ligands to HEK293T cells transfected with ZNRF3-ΔRING (ZNRF3 with deletion of the RING domain). Specific binding is calculated by subtracting background fluorescence (no ligand present) from the fluorescence reading at each ligand concentration. (**B**), Saturation binding of the indicated ligands with HEK293T cells transfected with RNF43ECD-TM. (**C**), TOPFlash activity of the indicated ligands in STF cells and STF-LGR4KO cells after normalization by cell number. Each graph is one representative of repeated experiments with each point being the mean of two replicates of the experiment. Error bars are S.E.M.

X-ray crystal structures of ECDs of RNF43 and ZNRF3 revealed that both ECDs exist as dimers and bind to RSPO furin domains in a 2:2 format^25^. In this configuration, the C-terminal ends of the two RSPO furin domains were only 43 Å apart and would be predicted to allow bivalent binding of RSPO furin domain. We compared the binding of R2Fu-mFc and -bFc on cells expressing RNF43-ECD anchored to the cell surface through its corresponding transmembrane domain (RNF43-ECDTM) or ZNRF3ΔRING which is inactive ZNFR3 due to deletion of the RING domain (over-expression of wild-type, active ZNRF3 is too toxic to the cells)^18^. The bivalent R2Fu-bFc displayed 6 × higher affinity on RNF43-ECDTM compared to R2Fu-mFc (1.1 ± 0.2 nM vs. 6.8 ± 1.8 nM) and 5 × higher on ZNRF3-ECDTM (2.7 ± 0.5 nM vs. 13.5 ± 3.3 nM), consistent with a dimeric nature of the E3 ligases (Fig. 4A,B and Supplementary Fig. S4A,B). We reasoned a lower affinity version of RSPO2 furin domain may reveal a larger difference between bivalent and monovalent binding. Thus, we expressed and purified R2Fu-bFc and -mFc with Gln70 to Arg (Q70R) mutation which was shown to decrease binding affinity of R2Fu to the E3 ligases^25^. As expected, bivalent R2Fu-Q70R-bFc showed much stronger binding to ZNRF3 when compared to the monovalent form (Kd of 2.9 ± nM vs. > 100 nM). Similar results were found with RNF43 (Kd of 6.3 ± 0.6 nM vs. > 100 nM). These results support the structural data that showed both RNF43/ZNRF3 exist as dimers that would permit bivalent binding of R2Fu-bFc on the cell surface.

We then compared activities of the bivalent and monovalent Q70R mutant in normal STF cells and STF-LGR4KO cells. R2Fu-bFc was active in both STF cells and STF-LGR4KO cells with much lower potency in LGR4 KO cells, consistent with the importance of LGR4 in increasing the affinity of RSPO binding to the E3 ligases. R2Fu-Q70R-bFc was active in STF cells albeit low potency and efficacy (Fig. 4C). In contrast, R2Fu-Q70R-mFc was completely inactive in STF-LGR4KO cells, though it was able to bind to RNF43 and ZNRF3 (Fig. 4A,B and C). These results suggest that the Gln70 residue is important in both binding and inhibiting the E3 ligase activity.

### Proposed models of LGR4, RSPO furin domain and E3 Ligase, and LGR5 and RSPO furin domain complexes on the cell surface

**Figure 5:**
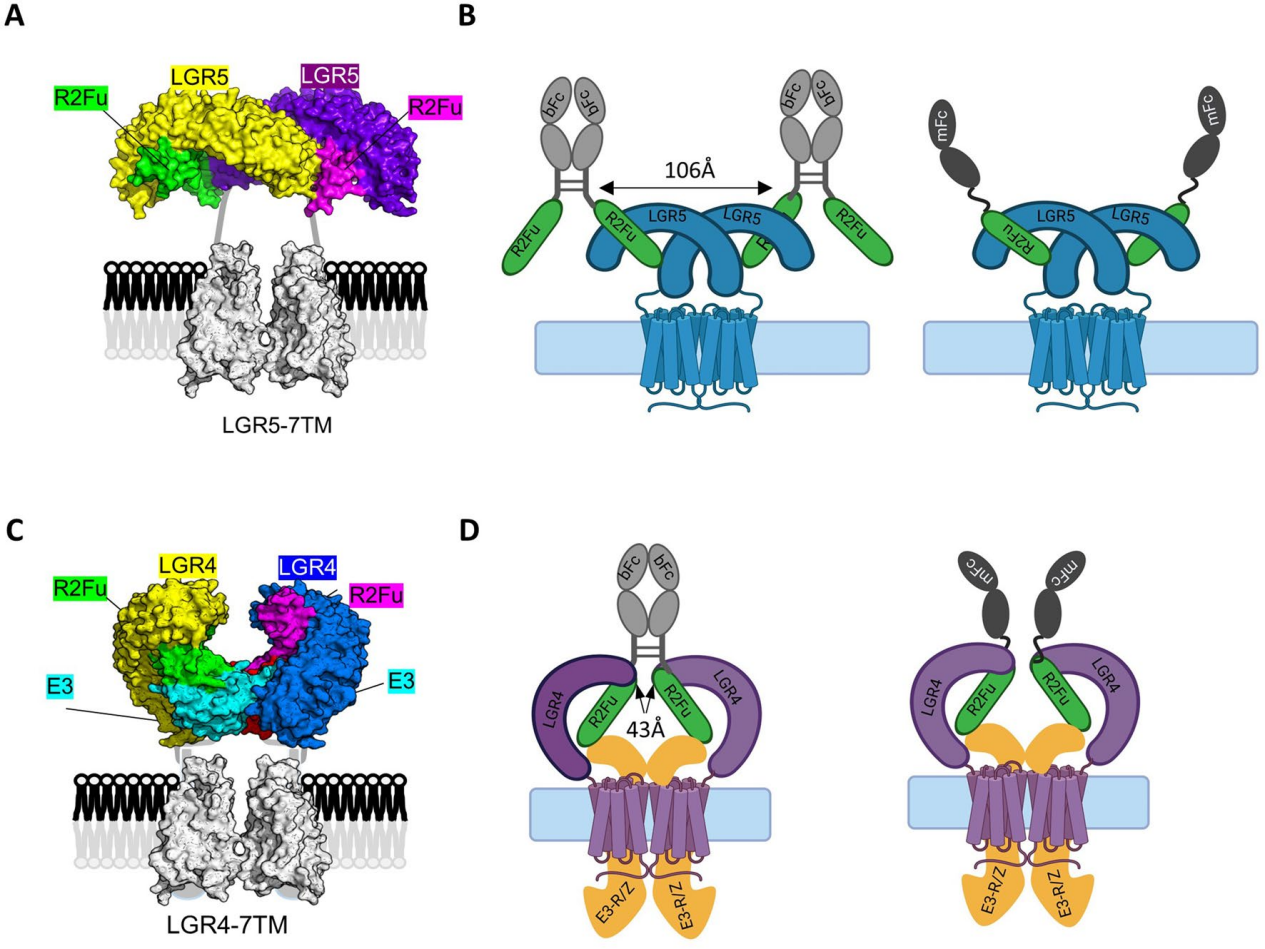
Structure models of RSPO furin binding to LGR4 and RSPO furin binding to the LGR4-E3 ligase complex on the cell surface. (**A**), Structure model of RSPO furin binding to LGR5 based on crystal structures of RSPO2 furin domain and LGR5ECD (PDB 4UFR) and rhodopsin 7TM (PDB 4J4Q). (**B**), Schematic diagram showing binding of bivalent versus monovalent RSPO furin domain to LGR5 cells. (**C**), Structure model of RSPO furin domain binding to LGR4-E3 ligase complex based on crystal structures of ZRNF3-RSPO2-FU (PDB 4C9E), LGR4ECD (PBD 4LI1), and rhodopsin 7TM (4J4Q). (**D**), Schematic diagram showing binding of bivalent and monovalent R2Fu-Fc to LGR4-E3 complex.

As full-length LGR5 expressed in whole cells does not interact with RNF43/ZNRF3 with or without RSPO, we proposed that the RSPO furin domain binds to LGR5 in a 2:2 configuration based on co-crystal structures of RSPO1/RSPO2 furin domain and LGR5ECD and rhodopsin 7TM (Fig. 5A)^24,26,36^. This is nearly identical to the structure model proposed by Peng et al. based on the X-ray co-crystal structures of RSPO1 furin domain and LGR5ECD^26^. In this configuration, the C-termini of the RSPO2 furin domain were 106 Å apart, too far for the Fc-based dimers to reach across, which is why bivalent and monovalent R2Fu had nearly identical affinity and the total amount of specific binding of bivalent R2Fu-bFc was approximately 2 × of the monovalent R2Fu-mFc (4 Fc domains vs. 2 Fc domains) (Fig. 2C). As discussed by Peng et al., in this configuration, the RSPO and LGR5 complex would be unable to bind to RNF43/ZRNF3-ECD due to steric hindrance imposed by the LGR5ECD *in trans*^26^.

Previously, we showed that full-length LGR4 forms a complex with full-length RNF43/ZNRF3 in whole cells even in the absence of RSPO^20^, which was also observed by Hao et al^18^. The crystal structures of ZNRF3- and RNF43-ECDs complexed with RSPO furin domain^25^, LGR4ECD complexed with RSPO1 furin domain^23^, and apo-LGR4ECD^23^ were all shown as 1:1 or 2:2 dimers^20,37^. Together with findings that LGR4 and RNF43/ZNRF3 all exist as dimers on the cell surface, we propose a model in which the LGR4 dimer encompasses the RNF43/ZNRF3 dimer in such a configuration that the ECDs of RNF43/ZNRF3 occupy the space above the seven transmembrane (7TM) domain of LGR4 (Fig. 5C). In this configuration, RSPO furin-1 domain binds to RNF43/ZNRF3 while furin-2 domain binds to LGR4 with the C-termini of furin-2 being only ~ 43 Å apart. This proximity allows for the two arms of bivalent R2Fu-bFc to bind the LGR4 and RNF43/RNF43 complex simultaneously. This model is consistent with the nearly equivalent maximum binding of R2Fu-bFc and R2Fu-mFc on cells expressing RNF43 or ZNRF3 as both forms would only have two Fc domains bound (Fig. 5D). This model is similar to the model proposed by Zebisch et al. for the LGR5-RSPO-E3 ligase heterotrimer complex^24^ except that full-length LGR5 expressed in whole cells does not interact with RNF43/ZNRF3 with or without RSPO^20^. The two models are consistent with the distinct profiles of RSPO binding to LGR4 and the E3 ligases as well as to LGR5 in whole cells.

### Monovalent RSPO furin domain had increased binding affinity in cells co-expressing LGR4 and ZNRF3 but not in cells co-expressing LGR5 and ZNRF3

**Figure 6:**
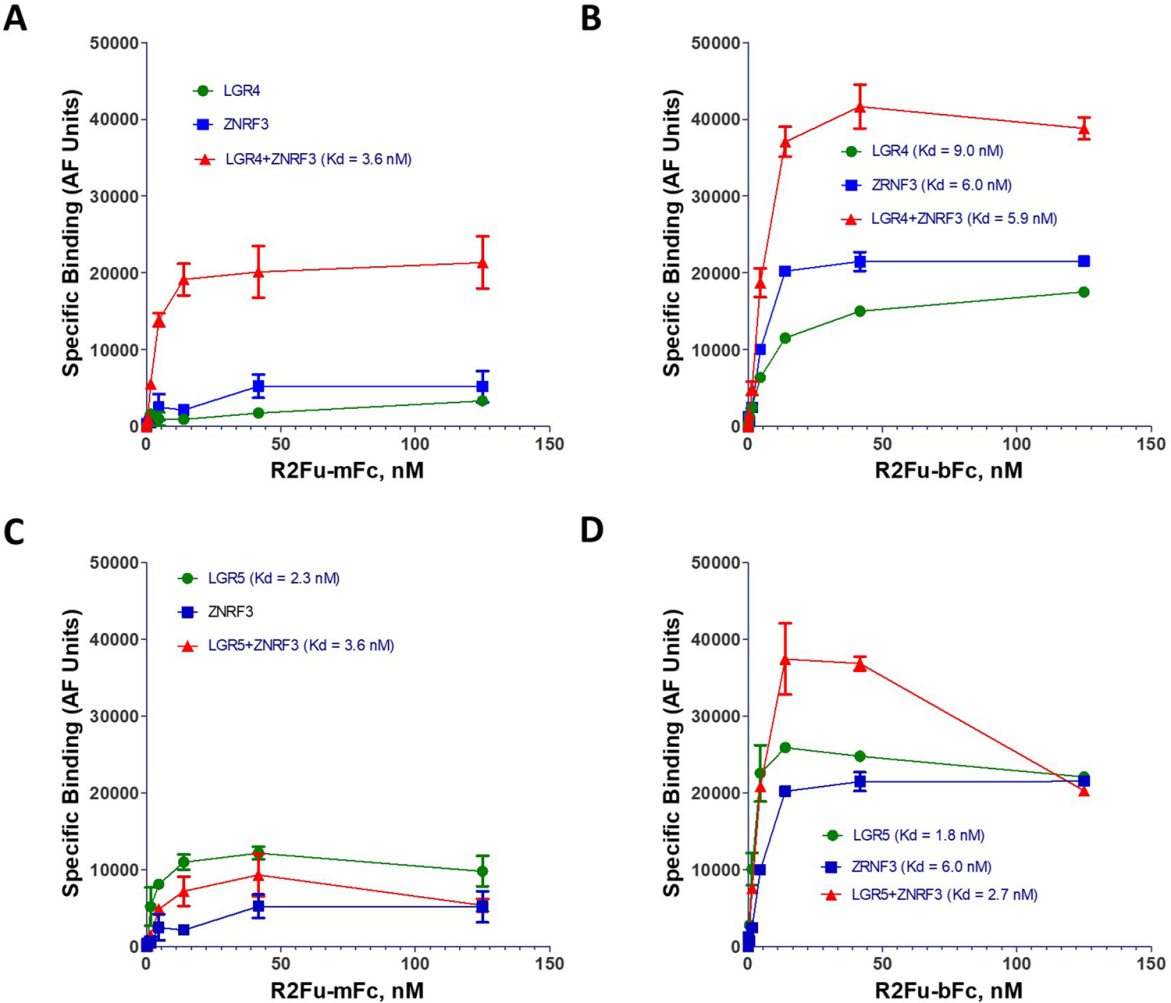
Co-expression of ZNRF3 with LGR4 but not with LGR5 increased binding affinity of monovalent RSPO furin domain. (**A**), Saturation binding of monovalent R2Fu to HEK293T cells over-expressing LGR4 or ZNRF3-ΔRING or both. (**B**), Saturation binding of bivalent of R2Fu to HEK293T cells over-expressing LGR4 or ZNRF3-ΔRING or both. (**C**), Saturation binding of monovalent R2Fu to HEK293T cells over-expressing LGR5 orZNRF3-ΔRING or both. (**D**), Saturation binding of bivalent R2Fu to HEK293T cells transfected with LGR5 or ZNRF3-ΔRING or both. For comparison, the ZNRF-ΔRING results were plotted in both (**A**) and (**C**), and (**B**) and (**D**). In all graphs, specific binding is calculated by subtracting background fluorescence (no ligand present) from total fluorescence. Each graph is one representative of repeated experiments with each data point being the mean of two replicates of that experiment. Error bars are S.E.M.

One of the crucial predictions of the model proposed in Fig. 5 is that co-expression of LGR4 and ZNRF3 would lead to significant increase in binding affinity of R2Fu-mFc but not that of R2Fu-bFc. To test this, we transfected ZRNF3ΔRING into HEK293T-vector cells or -LGR4 overexpressing cells and compared the binding affinity of R2Fu-bFc and -mFc. As shown in Fig. 6A (Supplementary Fig. S5A), co-expression of LGR4 and ZNRF3 led to increase in R2Fu-mFc binding in both affinity and total specific binding (Kd of 3.6 ± 0.9 nM vs. un-calculable). In contrast, co-expression of ZNRF3 and LGR4 had little impact on the binding of R2Fu-bFc (Fig. 6B and Supplementary Fig. S5B). For LGR5, co-expression of ZNRF3 had no effect on binding affinity of R2Fu-mFc, consistent with no interaction between the RSPO and LGR5 heterodimer complex and ZNRF3. R2Fu-bFc was not affected by co-expression of LGR5 and ZNRF3, as expected (Fig. 6C,D and Supplementary Fig. S5C,D). These results further suggest that the RSPO and LGR5 complex does not interact with the E3 ligases and high affinity binding of monovalent RSPO furin domain to the E3 ligases depends on co-expression of LGR4.

## Discussion

LGR4 and LGR5 are two related receptors with an overall sequence identity of ~ 50% and both receptors potentiate Wnt/β-catenin signaling in response to RSPOs^1,2,13,16^. Since the discovery that RSPO and LGR4 form a complex that binds and inhibits the activity of RNF43 and ZNRF3, it had been assumed that LGR4 and LGR5 mechanistically behave in a similar manner to potentiate Wnt/β-catenin signaling^22^. However, the two receptors have clearly been shown to have non-overlapping functions in organ development and stem cell survival, with LGR5 having a lesser role^13,15^. Previously, we showed that the RSPO and LGR5 complex does not interact with RNF43/ZNRF3 whereas LGR4 interacts with ZNRF3/ZNRF3, even without RSPO^20^. Here we have provided new evidence revealing that LGR4 and LGR5 display distinct configurations on the cell surface when probed by monovalent and bivalent ligands and proposed models that depict quaternary arrangements of LGR4 with RSPO and RNF43/ZNRF3 and of LGR5 with RSPO furin domain.

A major obstacle in understanding the signaling mechanism of the RSPO-LGR ligand receptor system is the lack of crystal structures of full-length receptors with and without ligands. Multiple crystal structures of LGR4ECD, LGR4ECD/RSPO-Fu, LGR5ECD/RSPO-Fu, LGR5ECD-RSPO-Fu-RNF43, and RSPO-Fu/RNF43ECD have been solved^21,23–26,38^, and the structure of LGR5ECD-RSPOFu-RNF43ECD heterotrimer was proposed as a general model for both LGR4 and LGR5. However, this model has not been validated in whole cells and in the absence of RNF43/ZNRF3-ECD, LGR5ECD and RSPO furin domain formed a 2:2 dimer that was incompatible with further binding to RNF43/ZRNF-ECD^26^. Similar to LGR4 and LGR5, we found that both RNF43 and ZNRF3 also exist as dimers on the cells surface, consistent with the X-ray structures of RNF43 and ZNRF3 in complex with RSPO furin domain that showed a 2:2 dimer architecture^25^. Combining this 2:2 complex of RNF43ECD and RSPO furin domain with the apo structure of LGR4ECD created a quaternary structure that would permit bivalent binding of RSPO furin domain, which is exactly what we observed when full-length LGR4 was co-expressed with ZNRF3 (Fig. 2C). In contrast, co-expression of ZNRF3 with LGR5 did not increase the potency of bivalent R2Fu when compared to the monovalent form. Interestingly, Chang et al. recently released a crystal structure of the Norrin ligand binding to LGR4ECD^39^. Norrin is a natural dimer and binds to LGR4ECD in a 2:2 form through the same region of LGR4ECD as the RSPO furin domain^39^. Remarkably, in the Norrin-LGR4ECD complex, the two LGR4 ECDs are positioned in the same way as the model we proposed here (Fig. 5B)^39^. Of note, Norrin does not appear to be able to make contact with RNF43/ZNRF3 in the LGR4-RNF43/ZNRF3 complex^39^. It would be interesting to test if Norrin binds to RNF43/ZNRF3, and if not, how Norrin enhances Wnt/β-catenin signaling through LGR4^40^.

In conclusion, we demonstrate that LGR4 and LGR5 displayed distinct profiles in RSPO binding in whole cells, and that RNF43 and ZNRF3 also existed as dimers on the cell surface. The LGR4 and RNF43/ZNRF3 complex accommodated bivalent binding of the R2Fu-bFc as predicted by the crystal structures of RSPO furin domain and ZNRF3ECD. LGR5, in contrast, adopts a dimer structure in which the two ECDs are oriented in a manner that does not allow for bivalent binding of R2Fu-bFc. These results reinforce the notion that LGR4 and LGR5 signals via different mechanisms in Wnt/β-catenin signaling and provide a glimpse of LGR4 and LGR5 configurations on the cell surface that has not been revealed by crystallography or cryo-EM analysis.

## Methods

### Plasmids and cloning

Expression plasmids encoding HA-LGR4, Myc-LGR5FL (full-length), Myc-LGR5ΔCT, HA-RNF43, and R2Fu-bFc were described previously^16,20,41,42^. Myc-ZNRF3ΔR was a gift from Dr. Feng Cong (Novartis Institute for Biomedical Research)^18^. R2Fu-His was constructed by cloning human RSPO2 furin domain (AA37-143) with an 8xHist tag into pCEP4. R2Fu-mFc was constructed with human RSPO2 furin domain (AA37-143) fused to monomeric human IgG4-Fc (CH2-CH3 domain CH2-CH3 with L351F, T366R, P395K, F405R and Y407E changes) using In-Fusion HD cloning^33^. All plasmids were verified by DNA sequencing.

### Recombinant proteins and purification of Fc-tagged R2Fu domain proteins

Recombinant R2FuTSP-His was purchased from R&D Systems. Purification of R2Fu-mFc and R2Fu-dFc were carried out by over-expressing in HEK293F cells followed by purification with protein-A beads as described previously^32^. In brief, 1 µg/mL DNA per 2.5 × 106 cells were transfected with 0.5 µg/µL polyethylenimine (PEI), and cells were supplemented with 2.2 mM valproic acid (VPA) 24 h post transfection. Supernatant was retrieved 8 days later, and target proteins were applied to a column with CaptivA protein A affinity resin (Repligen) for purification. Protein concentrations were determined using OD280, and purities were verified by Coomassie staining. His-tagged R2Fu was purified with Ni^2+^ beads following the beads manufacturer’s directions.

### Cell culture, transient transfection, and Wnt/β-catenin STF assay

HEK293-LGR4 and HEK293-LGR5ΔC, HEK293-STF, STF-LGR4KO, STF-LGR4KO-LGR5-overexpressing cells were generated and cultured as described before^16,20,30,32^. For transient transfection, ~ 80% confluent cells were transfected with DNA: FuGENE HD (Promega) ratio of 1:3 for all transient transfection presented. TOPFlash assays of HEK293-STF parental, STF-LGR4KO, and STF-LGR4KO-LGR5-overexpressing (oe) were carried out as described before^32^. All TOPFlash experiments were repeated at least once with duplicates or triplicates at each ligand concentration and signaling activity was normalized to cell number that was measured with the fluorescence dye alamar blue™.

### Antibodies and western blotting

For western blot analysis, anti-HA (Invitrogen cat #71-5500) and anti-β-actin (Cell Signaling cat #4970) were used. For ICC, Alexa 488-labeled anti-human IgG (Invitrogen cat #A11013) were used. Western blotting was carried as described previously^32^. In brief, cells were lysed with RIPA buffer (50 mM Tris–Cl pH 7.4, 150 mM NaCl, 1 mM DTT, 1% Triton X-100, 1% sodium deoxycholate, 0.1% SDS), supplemented with protease and phosphatase inhibitors, and reduced at 37 °C for 1 h. HRP-conjugated secondary rabbit or mouse antibodies (Cell Signaling) were used following the standard ECL protocol.

### Whole-cell binding analysis

Cells were seeded on the poly-D-lysine coated black, clear bottom 96-well plates, and cultured overnight. The plates were chilled on ice before binding assays started. His- or Fc-tagged RSPO2 furin domain proteins were diluted by 3 × serial dilution, added to the cell, and incubated on ice for 1 h. After washing and fixing of cells with 4.2% paraformaldehyde, cells were incubated with Alexa488-labeled anti-His antibody or Alexa555-labeled anti-human IgG antibody. Emission at 488 or 550 nm was measured using a plate reader (Tecan). Specific binding was calculated by subtracting background fluorescence (no ligand present, only fluorescence-labeled antibody) from total fluorescence signal. Dose–response curve (log(agonist) vs. response (three parameters)) were fitted using GraphPad Prism to retrieve half-maximum binding (K_D_). All experiments were repeated at least three times with duplicates or triplicates in each ligand concentration.

### Confocal immunofluorescence analysis

For binding of R2Fu-bFc and R2Fu-mFc to A549 and SK-N-AS cells, the cells were seeded in D-lysine-coated 8-chamber slides and cultured overnight. Ligands were added to final concentration of 125 nM and incubated at 37 °C with 95% humidity and 5% CO_2_ for 1 h, washed 3 × with PBS, and fixed with 4.2% paraformaldehyde, followed by permeabilization with 0.1% saponin. Then, the secondary antibody, anti-human-Alexa 488 was used to label Fc-tagged R2Fu. Nuclei were counterstained with TO-PRO-3. Cells were imaged under the Leica TCS SP5 confocal microscope and analyzed by the Leica LAS AF Lite software.

### Structure modelling

The structure model of RSPO2 furin and full-length LGR5 was generated by manually Rotate/Translate the crystal structures of RSPO2-Fu-LGR5ECD (PDB 4UFR) and rhodopsin 7TM (PDB 4J4Q) in COOT^43^. Structure model of LGR4-RSPO2 furin-E3 ligase was generated by superimposing RSPO2-FU structure in the crystal structures of LGR4ECD-RSPO2-FU (PBD 4KT1) onto the dimer of RSPO1-FU structure in the crystal structure of ZRNF3-RSPO1-FU (PDB 4C9E), and manually Rotate/Translate LGR4-RSPO2-E3 and rhodopsin 7TM in COOT. Cartoon and surface representations, distance measurement were generated with the PyMOL (http://www.pymol.org/).

### Data analysis and statistics

All data were analyzed using GraphPad Prism 5.

## Supplementary Information

Supplementary Information.

## Data Availability

The data used to support the findings of this study are available upon request from the corresponding author.
