# Supplementary material for: LGR4 and LGR5 form distinct homodimers that only LGR4 complexes with RNF43/ZNRF3 to provide high affinity binding of R-spondin ligands
Source: Sci Rep. 2023 Jul 4;13:10796. doi: 10.1038/s41598-023-37856-w (PMC10319729; doi:10.1038/s41598-023-37856-w)
Supplement: Supplementary file 1 — Supplementary Information. [file 41598_2023_37856_MOESM1_ESM.pdf]

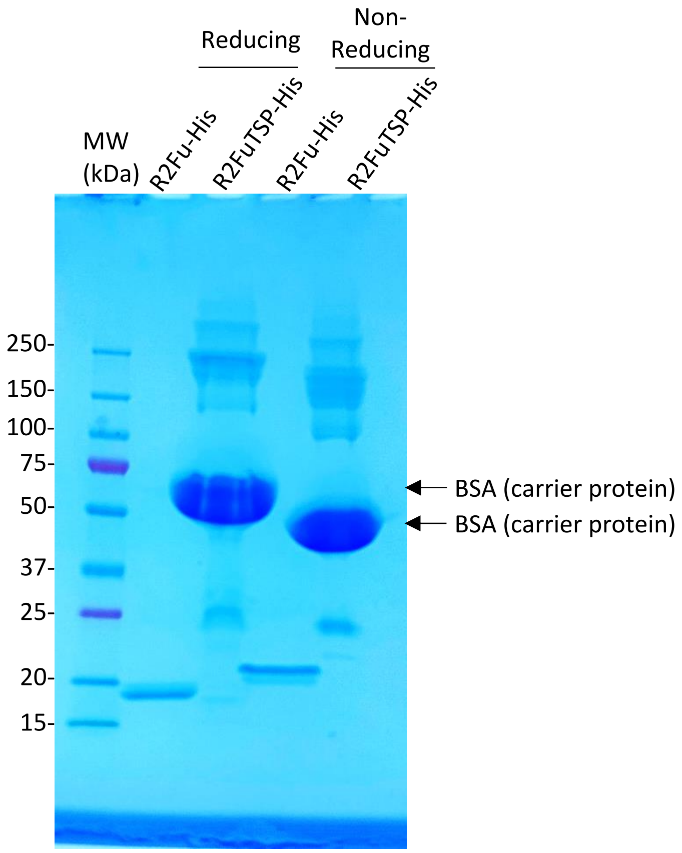

Supplementary Figure S1. Full gel image of the Coomassie blue staining of the SDS-PAGE cropped out in Figure 1B. The predicted molecular weight (MW) of R2Fu-His is 15 kDa and that of R2FuTSP-His is 22 kDa. The R2FuTSP-His was purchased from R&D systems and it contained BSA (bovine serum albumin) as a carrier protein.

A

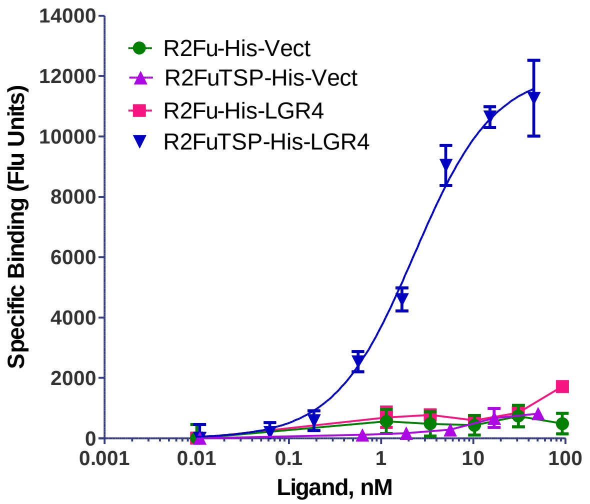

B

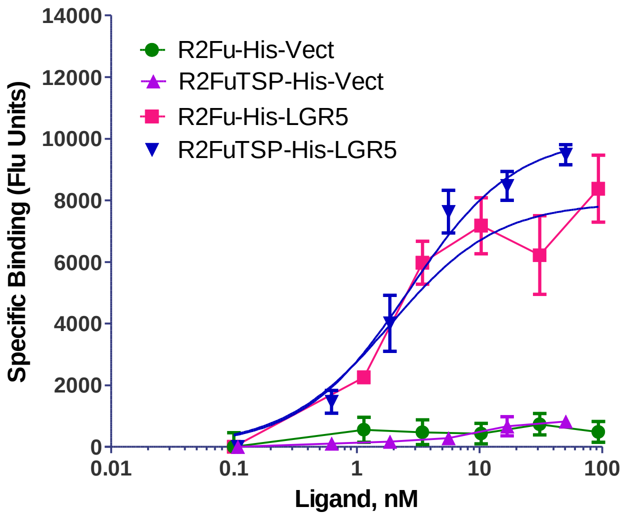

Supplementary Figure S2. Saturation binding of R2Fu-His and R2FuTSP-His to LGR4 and LGR5. A, the data of Figure 1D with the ligand concentration being plotted in a log scale. B, the data of Figure 1E with the ligand concentration being plotted in a log scale.

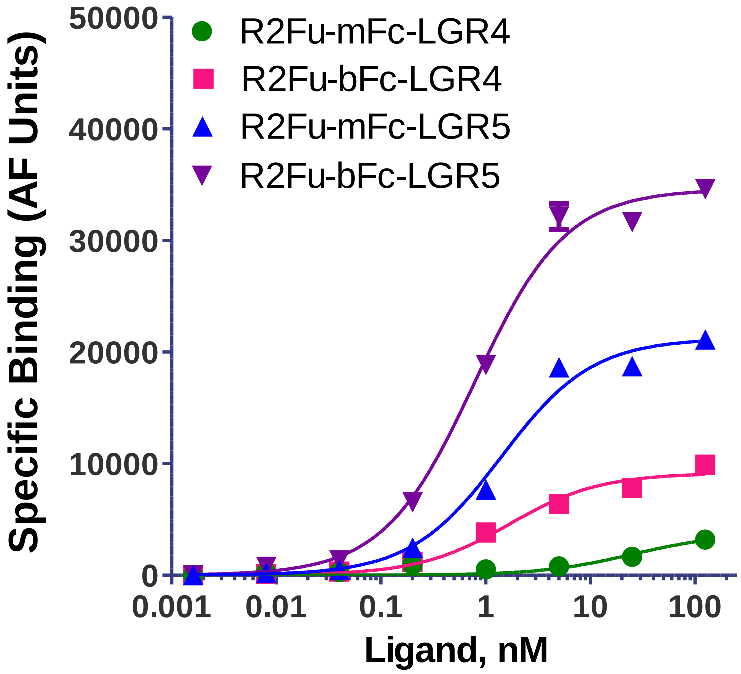

Supplementary Figure S3. Saturation binding of R2Fu-bFc and R2Fu-mFc to LGR4 and LGR5. This is from the data of Figure 2C except with the ligand concentration being plotted in a log scale.

A

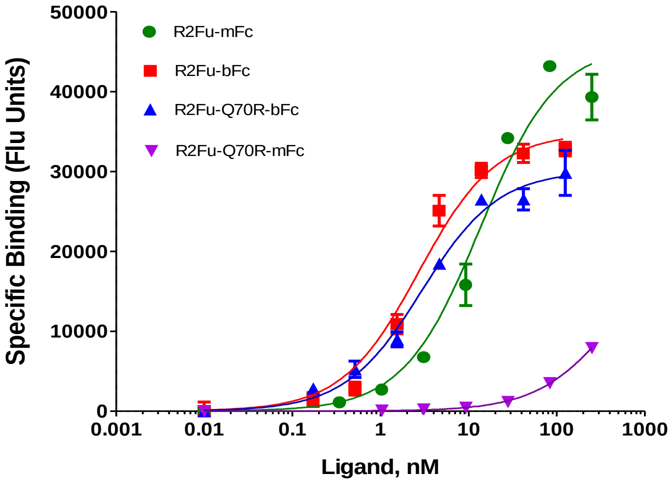

B

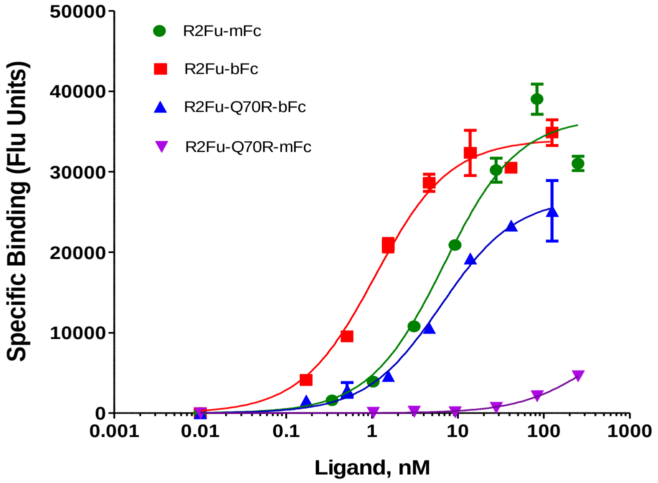

Supplementary Figure S4. Saturation binding of R2Fu-bFc and R2Fu-mFc to RNF43 and ZNRF3. A, the data of Figure 4A except with the ligand concentration being plotted in a log scale. B, the data of Figure 4B except with the ligand concentration being plotted in a log scale.

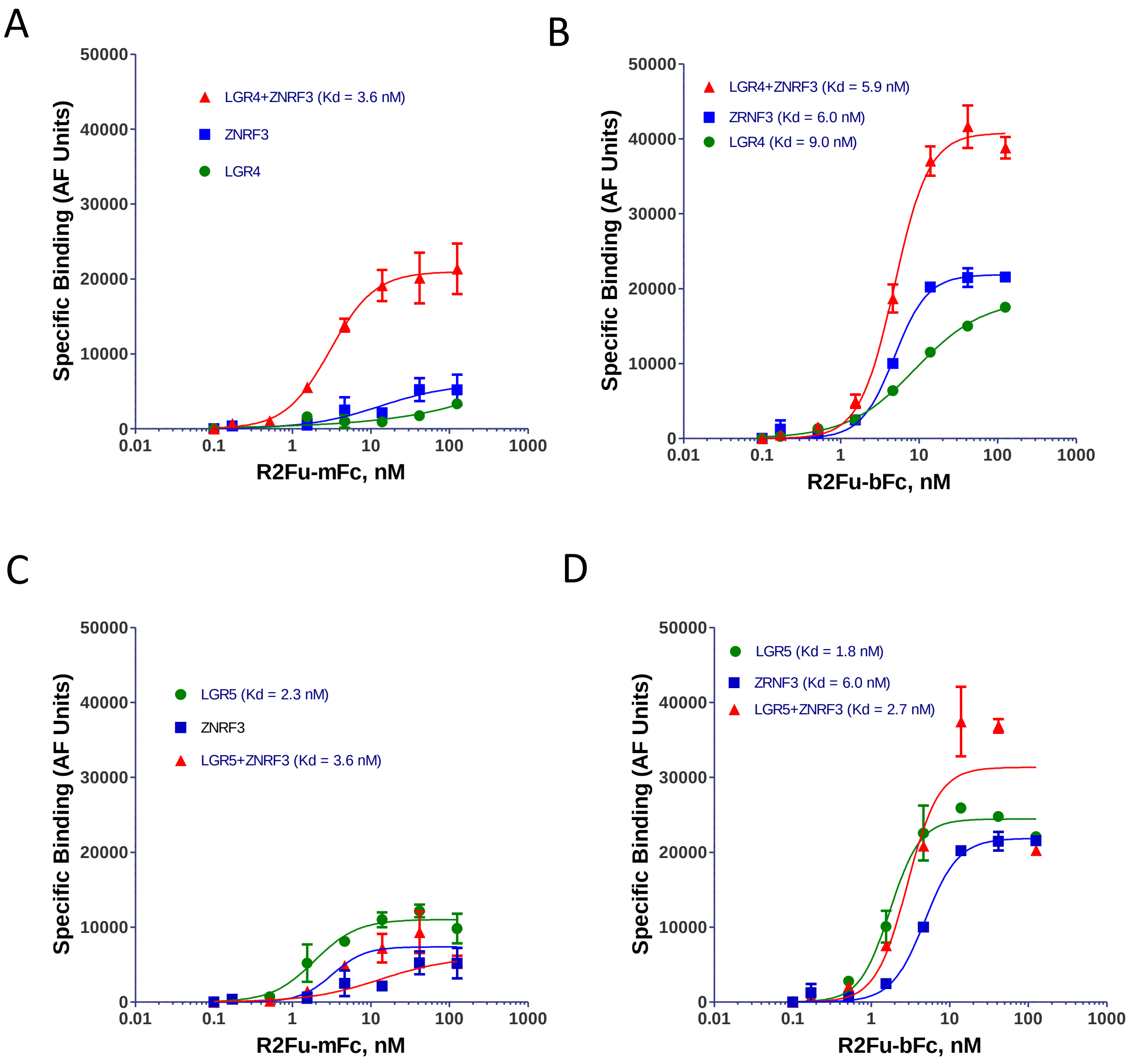

Supplementary Figure S5. Saturation binding of R2Fu-bFc and R2Fu-mFc to ZNRF3 with and without LGR4 or LGR5 co-expression. A-, the data of Figure 6 A-D except with the ligand concentration being plotted in a log scale.
